# Evaluating the Efficacy and Outcomes of Transcatheter Aortic Valve Replacement Versus Surgical Aortic Valve Replacement in Low‐ to Intermediate‐Risk Aortic Regurgitation Patients: A Comprehensive Clinical Comparison

**DOI:** 10.1155/crp/9978726

**Published:** 2026-04-13

**Authors:** Xiaoxue Zhang, Chenxi Yan, Shiliang Li, Cai Cheng

**Affiliations:** ^1^ Department of Cardiovascular Surgery, Tongji Medical College, Huazhong University of Science and Technology, Wuhan, 430030, Hubei, China, hust.edu.cn; ^2^ Department of Cardiovascular Surgery, The Second Affiliated Hospital, Zhejiang University School of Medicine, Hangzhou, 310000, Zhejiang, China, zju.edu.cn

**Keywords:** aortic valve regurgitation, care, surgical aortic valve replacement, transcatheter aortic valve replacement

## Abstract

**Background:**

Transcatheter aortic valve replacement (TAVR) has been established as an alternative to surgery for high‐risk aortic regurgitation (AR) patients. However, its applicability to low‐ and intermediate‐risk populations remains under investigation. This study evaluates the clinical outcomes, quality of care, and patient‐centered implications of TAVR versus surgical aortic valve replacement (SAVR) in this population.

**Methods:**

Between 2021 and 2024, clinical data were retrospectively analyzed from 70 AR patients at our center, including 37 who underwent TAVR and 33 who received SAVR with bioprosthetic valves. Baseline characteristics, perioperative metrics, and major clinical outcomes were assessed. International registry data (FRANCE‐TAVI and ALIGN‐AR) were utilized for external validity comparisons.

**Results:**

Baseline characteristics were comparable between groups. TAVR was associated with shorter operation time (*p* = 0.001), reduced blood use (*p* < 0.001), and shorter hospital stays (*p* = 0.021). No 30‐day mortality was observed in TAVR, whereas four deaths occurred in the SAVR group (*p* = 0.029). Conduction abnormalities differed, with complete left bundle branch block (CLBBB) more frequent in TAVR (*p* = 0.025) and complete right bundle branch block (CRBBB) in SAVR (*p* = 0.029). Despite its minimally invasive nature, ICU observation time remained similar (*p* = 0.339) due to perioperative complications. Economic analysis suggests potential cost savings with TAVR in specific scenarios.

**Conclusion:**

TAVR offers favorable short‐term outcomes for low‐ to intermediate‐risk AR patients, yet challenges in perioperative care require optimization. Long‐term studies and multicenter trials are needed to refine patient selection and postprocedural management strategies.

## 1. Introduction

Aortic regurgitation (AR) occurs due to abnormalities in the valve leaflets or the aortic root. Structural changes caused by congenital defects, degenerative processes, rheumatic fever, infection, or trauma, as well as aortic root dilation or disruption, can lead to AR. Surgical aortic valve replacement (SAVR), performed with extracorporeal circulation (ECC), has traditionally been the primary treatment for AR. However, ECC is associated with significant complications and high mortality rates. In the past decade, transcatheter aortic valve replacement (TAVR) has emerged as a less invasive alternative with promising outcomes [1].

Current guidelines from the European Society of Cardiology (ESC) and the European Association for Cardiothoracic Surgery (EACTS) have expanded the indications for TAVR in the treatment of aortic stenosis (AS) [2]. SAVR is recommended for younger, low‐risk patients (< 75 years, STS‐PROM or EuroSCORE II < 4%) or those ineligible for transfemoral TAVR. Meanwhile, TAVR is preferred for older patients (≥ 75 years) and those at high surgical risk (STS‐PROM/EuroSCORE II > 8%). For AR, however, the guidelines do not provide clear recommendations for TAVR. Despite this, several studies have demonstrated its safety and efficacy in high‐risk AR patients. The European Heart Survey found that up to 40% of patients with mixed aortic disease underwent TAVR with favorable clinical outcomes, supporting its potential use in inoperable AR [3–8].

Although clinical data on TAVR for low‐ and intermediate‐risk AR patients remain limited, its adoption is increasing worldwide. In China, where minimally invasive procedures are highly valued, the use of TAVR in these patient groups has risen significantly. This study aims to assess two key differences among low‐ and intermediate‐risk AR patients: (1) clinical outcomes and complications between TAVR and SAVR and (2) differences in care management.

This study also evaluates the comparative effectiveness of TAVR versus SAVR in low‐ to intermediate‐risk AR patients and discusses the broader implications for healthcare quality, patient experience, and cost‐effectiveness.

## 2. Methods

This retrospective clinical study was conducted at Tongji Hospital, Tongji Medical College, Huazhong University of Science and Technology. Low to moderate surgical risk was defined as age < 80 years and EuroSCORE < 8%. Data were retrospectively collected through electronic medical record (EMR) review and manual chart extraction. Two independent researchers verified the data accuracy (ZX and CX).

### 2.1. Patient Selection

Inclusion criteria were as follows: (1) age between 18 and 80 years; (2) diagnosis of moderate‐to‐severe AR confirmed by echocardiography; (3) presence of significant symptoms; (4) selection of SAVR with a bioprosthetic valve; and (5) EuroSCORE < 8%.

Exclusion criteria were as follows: (1) history of previous heart valve surgery; (2) incomplete clinical data; (3) presence of AS or calcification; and (4) diagnosis of infective endocarditis, rheumatic heart disease, or aortic valve bicuspid malformation.

**FIGURE 1:**
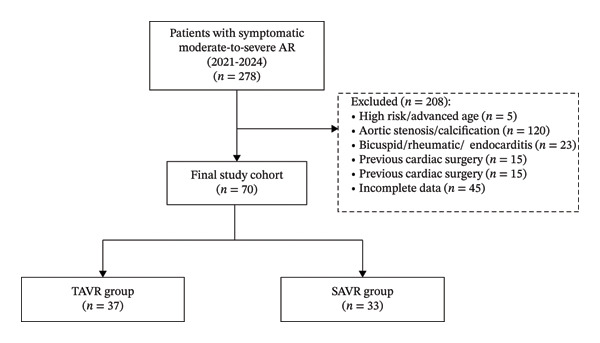
Study flowchart.

Between 2021 and 2024, 70 patients met the inclusion criteria. Among them, 37 underwent TAVR and 33 underwent SAVR. Clinical data were collected at baseline, perioperatively, and 30 days postoperatively (Figure 1).

### 2.2. Indication for Procedure Choice

The final treatment strategy (TAVR or SAVR) for each patient was determined by a multidisciplinary heart team consisting of cardiac surgeons, interventional cardiologists, and anesthesiologists. The decision‐making process was based on a comprehensive evaluation of anatomical suitability (e.g., aortic annulus dimensions, aortic root geometry, and peripheral vascular access), surgical risk (EuroSCORE II), and patient preference. Generally, TAVR was considered for patients with suitable femoral access and anatomical features favorable for device anchoring, whereas SAVR was recommended for patients with anatomical contraindications to TAVR or those requiring concomitant cardiac procedures.

### 2.3. Definition

Primary clinical outcomes were defined according to the Valve Academic Research Consortium (VARC‐3) criteria. The VARC‐3 criteria define the primary endpoint as composite event outcomes of 30‐day all‐cause mortality, all‐cause stroke (disabling and nondisabling), hemorrhage (VARC type 3 and 4 bleeding), acute kidney injury (grades 2–4), major vascular complications, moderate or severe prosthetic valve regurgitation (grade II or III), and conduction system disorders resulting in the need for permanent pacemaker implantation (PPI). The 1‐year rehospitalization was also added as one of the primary clinical outcomes.

Perioperative data included operative time, intraoperative red blood cell transfusion, life‐threatening hemorrhage, second valve implantation, delirium, mild pericardial effusion, severe postoperative heart failure, and so on (details given in Table 1). Delirium is assessed according to the Confusion Assessment Method (CAM) and is diagnosed if 1 + 2 of the following conditions are met and at least 3 or 4 of the following conditions are met: (1) sudden onset and fluctuating course; (2) inattention; (3) disorganized thinking; and (4) altered level of consciousness. Mild pericardial effusion was defined as a diastolic effusion thickness of < 10 mm and an estimated effusion volume of < 100 mL, as assessed by echocardiography. Postoperative heart failure was diagnosed based on the presence of at least one of the following criteria: (1) significant worsening of symptoms, including orthopnea, pink frothy sputum, peripheral or generalized edema, or reduced urine output; (2) BNP > 400 pg/mL or NT‐proBNP > 900 pg/mL beyond 5 days postoperatively; (3) left ventricular ejection fraction (LVEF) < 40%; (4) cardiac index < 2.2 L/min/m^2^; (5) central venous pressure (CVP) > 10 mmHg; or (6) chest X‐ray findings suggestive of pulmonary congestion, including pulmonary hematoma, pulmonary edema (Kerley B lines and butterfly pattern), or cardiomegaly (cardiothoracic ratio > 0.5). Severe postoperative heart failure was defined as the presence of any two or more of these criteria.

**TABLE 1:** Comparison of perioperative data between TAVR group and SAVR group.

Data	TAVR group [*n* = 37 (53%)]	SAVR group [*n* = 33 (47%)]	*p* value	Difference (95% CI)
Operation time [mid (min–max), min]	239 (121–554)	310 (165–810)	0.001	−69.3 (−120.1∼−18.5)
Intraoperative red cell transfusion [mid (min–max), U]	0	2 (0–13.5)	< 0.001	−2.0 (−3.1∼−0.9)
Intraoperative life‐threatening hemorrhage [*n* (%)]	0	3 (9)	0.199	−9.1 (−18.9∼0.7)
Second valve implantation [*n* (%)]	2 (5)	0	0.494	5.4 (−1.9∼12.7)
Permanent pacemaker implantation [*n* (%)]	2 (5)	1 (3)	1.000	2.4 (−7.0∼11.7)
IABP [*n* (%)]	0	2 (6)	0.219	−6.1 (−14.2∼2.1)
Postoperative severe heart failure [*n* (%)]	0	6 (18)	0.022	−18.2 (−31.3∼−5.0)
Postoperative ICU time [mid (min–max), d]	2 (1–8)	2 (1–8)	0.339	0.6 (−0.3∼1.4)
Postoperative hospital stay [mid (min–max), d]	10.5 ± 4.6	13.5 ± 5.8	0.021	−2.9 (−5.5∼−0.4)
LVEF before discharge from hospital [mid (min–max), %]	56 (35–68)	57 (22–73)	0.823	−0.5 (−5.1∼4.1)
Duration of mechanical ventilation [median (IQR), h]	1.5 (0.5–3.0)	9.0 (6.0–14.5)	< 0.001	−8.2 (−10.5∼−5.9)
Postoperative complications [*n* (%)]				
Postoperative infection	1 (2.7)	2 (6.1)	0.598	−3.4 (−13.0∼6.3)
Mild pericardial effusion	13 (35)	19 (58)	0.060	−22.4 (−45.3∼0.4)
New or worsening atrial fibrillation (AF)	3 (8)	3 (9)	1.000	−1.0 (−14.2∼12.2)
III degree AV block	3 (8)	0	0.280	8.1 (−0.7∼16.9)
Complete left bundle branch block (CLBBB)	7 (19)	0	0.025	18.9 (6.3∼31.5)
Complete right bundle branch block (CRBBB)	0	4 (12)	0.029	−12.1 (−23.3∼−1.0)
Delirium	0	7 (21)	0.011	−21.2 (−35.2∼−7.3)

### 2.4. Procedural Characteristics

All procedures in this study were performed on an elective basis. For the TAVR group, the transfemoral approach was the primary access route. All TAVR patients received self‐expanding valve systems, which were chosen for their superior anchoring capabilities in noncalcified aortic valves. The procedures were performed under general anesthesia or local anesthesia with conscious sedation, depending on clinical judgment. In the SAVR group, all patients underwent valve replacement with bioprosthetic valves via median sternotomy under general anesthesia and cardiopulmonary bypass.

### 2.5. Statistical Analysis

Statistical analyses were conducted using SPSS (Version 26.0). Continuous variables with a normal distribution were reported as mean ± standard deviation, while nonnormally distributed continuous variables were expressed as mid (min–max). Categorical variables were presented as counts and percentages [*n* (%)]. Comparisons between normally distributed continuous variables were performed using the two‐sample *t*‐test, while nonnormally distributed continuous variables were analyzed using the nonparametric rank‐sum test. Unordered categorical variables were compared using the chi‐square test, and ordered categorical variables were analyzed using the nonparametric rank‐sum test. A *p* value < 0.05 was considered statistically significant.

Power analysis estimated a required sample size of 90 patients (45 per group) to detect a 10% difference in major adverse events with 80% power at a significance level of 0.05. Our study (*N* = 70) suggests a moderate power to detect significant clinical differences. Sensitivity analysis was performed, excluding early mortality cases.

## 3. Results

### 3.1. Baseline Data

Table 2 presents the baseline characteristics of both patient groups, including age, gender, BMI, EuroSCORE II, NYHA classification, LVEF, eGFR, and comorbidities such as hypertension, diabetes, and stroke. The groups were well‐matched, with no significant differences between them (*p* > 0.05), ensuring comparability in clinical outcomes. Notably, a higher proportion of male patients were observed in the AR group.

**TABLE 2:** Comparison of baseline date between TAVR group and SAVR group.

Baseline data	TAVR group [*n* = 37 (53%)]	SAVR group [*n* = 33 (47%)]	*p* value
Age [mid (min–max), year]	64 (36–78)	64 (39–74)	0.489
Sex (male/female, *n*)	26/11	27/6	0.261
BMI [mid (min–max), kg/m^2^]	24 (19–33)	24 (18–33)	0.997
EuroSCORE II [mid (min–max), %]	2.1 (0.8–6.5)	1.9 (0.9–3.9)	0.935
NYHA classification ≥ III [*n* (%)]	19 (51)	19 (58)	0.602
LVEF [mid (min–max), %]	63 (35–74)	62 (39–75)	0.184
eGFR [mid (min–max), mL/(min·1.73 m^2^)]	82.5 (5.7–101.4)	83.4 (4.3–107.0)	0.952
Hypertension [*n* (%)]	14 (38)	19 (58)	0.099
Diabetes [*n* (%)]	5 (14)	2 (6)	0.523
Stroke [*n* (%)]	5 (14)	5 (15)	1.000

### 3.2. Perioperative Data

We compared key perioperative metrics between the two groups from surgery to hospital discharge (Table 1). Operation time (*p* = 0.001), intraoperative blood use (*p* < 0.001), and postoperative hospital stay (*p* = 0.021) were significantly lower in the TAVR group than in the SAVR group. However, postoperative ICU observation time did not differ significantly (*p* = 0.339). Postoperative severe heart failure occurred in 18% of SAVR patients, while no cases were observed in the TAVR group, a significant difference (*p* = 0.022). Additionally, conduction abnormalities varied between groups: complete left bundle branch block (CLBBB) was more common in the TAVR group, while complete right bundle branch block (CRBBB) predominated in the SAVR group (*p* = 0.025, *p* = 0.029). The duration of mechanical ventilation was significantly shorter in the TAVR group compared to the SAVR group (median 1.5 (0.5–3.0) h vs. 9.0 (6.0–14.5) h, *p* < 0.001). Notably, no patients in the TAVR group developed postoperative delirium, whereas 21% of SAVR patients did—a significant difference (*p* = 0.011). Other postoperative complications, including the incidence of postoperative infection, mild pericardial effusion, new‐onset atrial fibrillation, and III atrioventricular block, showed no significant differences between groups (*p* > 0.05). Similarly, rates of intraoperative life‐threatening hemorrhage, second valve implantation, PPI, and intra‐aortic balloon pump (IABP) use did not differ significantly (*p* > 0.05). Cardiac ultrasound performed before discharge showed no significant difference in LVEF between the two groups.

### 3.3. Primary Clinical Outcomes

**FIGURE 2:**
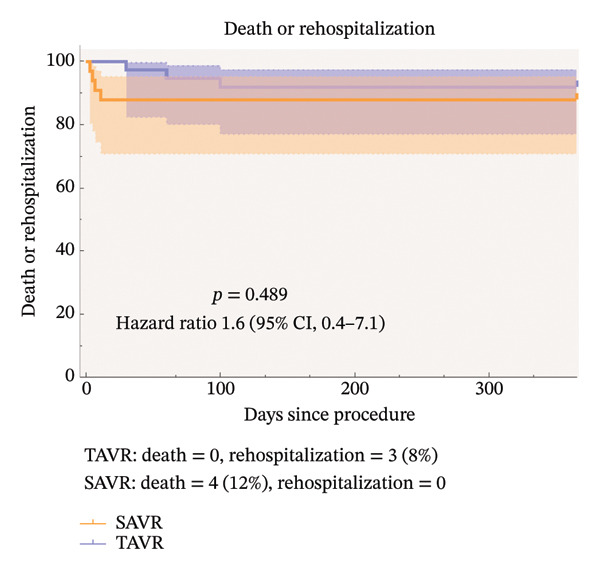
Time‐to‐event curves for death or rehospitalization.

**FIGURE 3:**
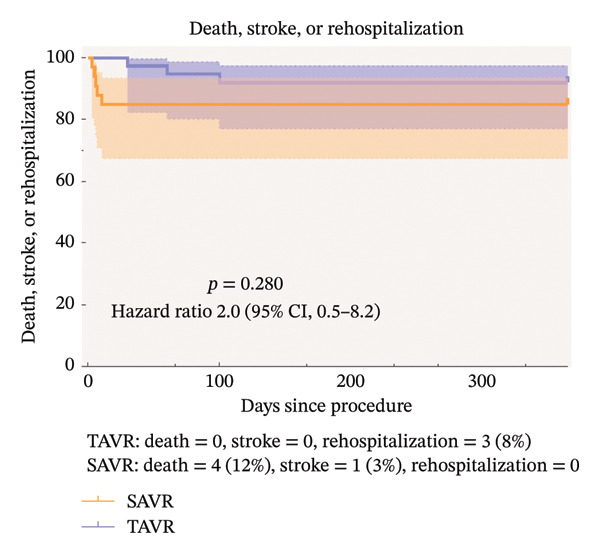
Time‐to‐event curves for death, stroke, or rehospitalization.

As shown in Table 3, no patients in the TAVR group experienced fatal events within 30 days postoperatively, while 4 deaths due to cardiovascular events occurred in the SAVR group, though the difference was statistically significant (*p* = 0.029). Stroke was reported in 1 SAVR patient (*p* = 0.471). Three SAVR patients experienced major hemorrhage, while none occurred in the TAVR group (*p* = 0.199). Postoperative renal insufficiency was common in both groups, with no significant difference in incidence (*p* = 0.937). Additionally, 2 SAVR patients developed intraoperative aortic oozing. At 30 days postoperatively, no patients in either group had moderate to severe AR. Three patients in the TAVR group were readmitted to the hospital within 1 year after procedure for mitral valve malfunction and coronary artery disease, respectively. Defining the endpoint event as death or rehospitalization and the time as the number of days after procedure to the occurrence of the endpoint event, it can be observed in Figure 2 that the risk of endpoint events was similar for TAVR and SAVR (*p* = 0.489, 95% CI 0.4–7.1). After we added stroke, the risk of endpoint events was still similar for TAVR and SAVR (Figure 3).

**TABLE 3:** Comparison of major clinical outcomes between TAVR group and SAVR group.

Data	TAVR group [*n* = 37 (53%)]	SAVR group [*n* = 33 (47%)]	*p* value	Difference (95% CI)
Mortality [*n* (%)]				
30‐day all‐cause	0	4 (12)	0.029	−12.1 (−23.3∼−1.0)
30‐day cardiovascular	0	4 (12)	0.029	−12.1 (−23.3∼−1.0)
Major stroke (30 days) [*n* (%)]	0	1 (3)	0.471	−3.0 (−8.9∼2.8)
Hemorrhage [*n* (%)]	0	3 (9)	0.199	−9.1 (−18.9∼0.7)
Acute kidney injuries [*n* (%)]	7 (19)	6 (13)	0.937	0.7 (−17.5∼19.0)
Vascular complications [*n* (%)]	0	2 (6)	0.219	−6.1 (−14.2∼2.1)
Moderate/severe prosthetic valve regurgitation [*n* (%)]	0	0	—	—
Permanent pacemaker implantation [*n* (%)]	2 (5)	1 (3)	1.000	2.4 (−7.0∼11.7)

### 3.4. Comparison to International Registries

Our findings align with FRANCE‐TAVI (2015–2021) and ALIGN‐AR (2018–2022), which report reduced mortality and shorter hospital stays with TAVR, even in intermediate‐risk patients. However, our study population had lower EuroSCORE values, emphasizing the need for tailored risk stratification.

## 4. Discussion

This study demonstrates that for low‐ to intermediate‐risk patients with pure AR, TAVR offers a viable alternative to SAVR with distinct perioperative advantages and comparable short‐term clinical outcomes. It was observed that TAVR significantly reduced operative time, intraoperative blood utilization, and the duration of mechanical ventilation compared to SAVR. However, this minimally invasive benefit did not translate into a shorter ICU observation time, likely due to the need for arrhythmia monitoring. Regarding postoperative complications, distinct profiles were identified: SAVR was associated with a higher incidence of severe postoperative heart failure and delirium, whereas TAVR was more frequently associated with conduction disturbances, specifically CLBBB. Despite these differences, major clinical outcomes, including stroke and rehospitalization, remained comparable between the two groups. Notably, a higher proportion of male patients were observed in this cohort, potentially reflecting a gender‐specific prevalence in this specific population.

The findings indicate that the minimally invasive benefits of TAVR were not reflected in postoperative ICU observation time. Although TAVR involves less invasive surgery, patients remain susceptible to arrhythmias, necessitating ICU monitoring comparable to that of SAVR. Additionally, the recovery of cardiac function after TAVR did not show a significant advantage. It is hypothesized that the prosthetic valve may have caused myocardial and conduction bundle damage during implantation, or that the valve size was relatively large—a common requirement in AR patients to ensure radial force fixation. TAVR is also associated with postoperative bleeding from the femoral artery due to the access incision. For instance, one female patient with preoperative thrombocytopenia developed two femoral artery bleeds following TAVR, leading to an extended ICU stay. Several factors were identified contributing to prolonged ICU observation after TAVR in this population: (1) femoral artery bleeding, (2) difficult‐to‐control arrhythmias, and (3) slower recovery of cardiac function. In contrast, prolonged ICU observation after SAVR was primarily attributed to sternal misalignment, severe heart failure, wound bleeding, and severe delirium.

Postoperative delirium was infrequent in the TAVR group, which can be primarily attributed to two factors: the relatively younger age of the patients and the shorter duration of the procedure, including reduced anesthesia time. These factors may contribute to a quicker recovery of cognitive function and reduced susceptibility to delirium. Conversely, despite similar age profiles, seven patients in the SAVR group developed severe delirium postoperatively. This disparity suggests that the extended surgical time and more invasive nature of SAVR may increase the risk of postoperative cognitive disturbances, underscoring the importance of targeted delirium prevention strategies in invasive procedures.

Distinct arrhythmia patterns were also identified between the groups, with CLBBB being more prevalent after TAVR and CRBBB more common after SAVR. These differences likely result from varying intraoperative mechanisms of mechanical injury and the unique anatomical locations of the cardiac conduction system. The left bundle branch (LBB), positioned near the membranous septum and aortic annulus, is particularly susceptible to direct mechanical trauma during TAVR stent expansion. Conversely, the right bundle branch (RBB) is less exposed to TAVR pressure but is vulnerable during SAVR due to aortic annulus debridement, suturing, and ischemic injury during cardioplegic arrest. CRBBB is generally well tolerated, whereas CLBBB can impair ventricular function. These findings highlight the need for tailored postoperative strategies. The distinct patterns of arrhythmia observed necessitate individualized management approaches, as outlined in Table 4.

**TABLE 4:** The key management strategies of different types of bundle branch block between TAVR and SAVR.

Management content	Measure	
	TAVR (CLBBB)	SAVR (CRBBB)
Prevention	Limiting valve implantation depth, reducing membrane septal compression.	Intraoperatively protect the right bundle branch and avoid deep sutures.

Monitoring	Postoperative 48–72 h ECG monitoring, alert for PR prolongation + LBBB.	Postoperative 72 h cardiac monitoring for RBBB progression.

Treatment	1. Mild LBBB: observation + beta‐blocker modulation; 2. With PR prolongation or further QRS widening: consider reimplantation of a temporary pacemaker.	1. Mild RBBB: observation + beta‐blocker modulation; 2. If combined with PR prolongation or triple‐bundle branch block: consider pacemaker.

Upon reviewing the management approach for AR patients, several areas require further reflection. Although TAVR avoids pericardiotomy and should theoretically reduce pericardial effusion, no significant difference was revealed in the incidence of mild pericardial effusion compared to SAVR. This discrepancy implies two perspectives. Intraoperatively, it may stem from minor damage to cardiac structures during valve deployment or annular leakage due to valve overexpansion. Postoperatively, factors such as insufficient monitoring, prolonged bed rest hindering absorption, or fluid retention may contribute. Given the rapid recovery in younger, low‐risk patients, there may be a tendency to overlook subtle postoperative signs. However, neglected pericardial effusion can lead to tamponade. Therefore, postoperative management of pericardial effusion should be rigorously maintained in TAVR patients. Recommended management strategies are summarized in Table 5.

**TABLE 5:** The management of pericardial effusion between TAVR and SAVR.

Manage content	TAVR	SAVR
Material cause	The valve expansion process damaged the heart perforation and the membrane ventricular septum.	Surgical incision, pericarditis, and sternal suture affected the pericardial fluid absorption.
Preventive measure	Precise valve positioning to avoid low‐level implantation; intraoperative ultrasound monitoring.	Strict control of intraoperative bleeding to reduce the postoperative inflammatory response.
Postoperative monitoring	Echocardiographic evaluation at 24–48 h after surgery.	The postoperative pericardial drain was maintained for 48–72 h and the drainage rate was monitored.
Treatment	Small volume of effusion: close monitoring, diuretic control. Mass effusion: pericardiocentesis drainage.	Pleural drainage: if excessive fluid accumulation requires ultrasound‐guided pericardiocentesis.

It was observed in a comprehensive review of centers across China that an increasing number of institutions are extending TAVR to patients under 80 years of age with low surgical risk. This reflects a global trend supported by international data [9]. For instance, Oettinger et al. highlighted TAVR’s potential as a more efficient option [10], and the FRANCE‐TAVI registry concluded that TAVR was safe even in patients with complex pathologies [11]. Furthermore, the ALIGN‐AR pilot study reported a mean STS‐PROM score of 4.1% for AR patients treated with TAVR [12], and A Rocha De Almeida et al. found TAVR to be effective in patients younger than 75 years [13]. Cultural and psychological factors, including patient preference for minimally invasive options, play a significant role in this shift. Specifically, for patients aged < 50 years in this study cohort, the choice of bioprosthesis or TAVR was often based on specific contraindications to anticoagulation, childbearing potential, or strong patient preference after detailed consultation regarding valve durability.

Given these developments, it is essential to consider how care strategies differ. Based on clinical experience, tailored preoperative and postoperative protocols have been refined (Table 6). For TAVR, assessments focus on vascular access and conduction risk, whereas SAVR evaluations address surgical tolerance. The distinct recovery trajectories—rapid physical recovery with TAVR versus the need for robust pain management and wound care in SAVR—highlight the importance of customizing nursing and medical management to optimize outcomes.

**TABLE 6:** Differences in the care and management of low‐ to moderate‐risk young AR patients treated with TAVR and SAVR.

Phase	Manage content	TAVR	SAVR
Preoperative	Anatomical assessment	CT evaluated the aortic valve anatomy, valve ring diameter, and calcification and screened the patients suitable for TAVR.	Preoperative echocardiography and CT evaluated the aortic valve and ascending aorta to determine the suitability of the valve ring suture.
	Surgical risk assessment	To mainly assess vascular access feasibility, valve matching, and atrioventricular conduction risk.	To assess surgical tolerance, cardiopulmonary function reserve.
	Anticoagulation strategy	Warfarin was stopped before surgery or the antiplatelet regimen was adjusted, and intraoperative heparinization was performed.	Anticoagulants were stopped before surgery and routinely heparinized during surgery.
	Preoperative infection prevention	Preoperative antibiotics to prevent the infection.	Preoperative antibiotics, focusing on the risk of postoperative incision infection.
	Patient selection	It is suitable for patients with no severe valve ring calcification and suitable aortic valve anatomy.	It is suitable for young patients with large rings and needing long‐term valve durability assurance.

Postoperative	Postoperative analgesic management	Mild pain, mainly with oral analgesics.	Post‐thoracotomy pain is significant and requires a PCA analgesic pump or opioid analgesia.
	Heart function monitoring	CLBBB progression was followed and ECG was monitored for at least 48 h.	Concern for CRBBB or complete AV block with ECG monitoring for at least 72 h
	Anticoagulation management	Dual antiplatelet therapy (aspirin + clopidogrel) maintained for 3–6 months after procedure.	Bioflaps usually do not require long‐term anticoagulation but do require short‐term (3–6 months) antiplatelet therapy.
	Activity resume	Bedside activities are allowed 6–12 h after procedure, and get out of bed within 24 h.	Postoperative 48–72 h gradual return to activities, chest belt fixed for 3 months, prohibit thoracic movement to avoid sternal displacement.

### 4.1. Economic and Healthcare Policy Implications

The financial burden of heart valve replacement therapies is a major consideration for healthcare systems worldwide. Although TAVR reduces perioperative morbidity and hospital stay, the upfront device cost remains significantly higher than that of SAVR. Several cost‐effectiveness studies have attempted to determine whether these benefits translate into long‐term healthcare savings. A cost–utility analysis in the Netherlands demonstrated that TAVR generated 0.89 additional quality‐adjusted life years (QALYs) compared to SAVR at an incremental cost of €4742 per patient, leading to an incremental cost‐effectiveness ratio (ICER) of €5346 per QALY gained [14]. This ICER is within the widely accepted cost‐effectiveness threshold of €50,000 per QALY in Europe, suggesting that TAVR is a viable economic alternative for low‐risk patients. Similarly, a French model–based cost‐effectiveness analysis concluded that TAVR was cost‐effective in 74.4% of probabilistic sensitivity analyses, reinforcing its economic viability in patients at low surgical risk [15]. These findings suggest that, despite the higher initial cost, TAVR’s reduced hospital stay and post‐discharge care requirements may balance overall expenses over time.

Beyond initial procedural costs, long‐term healthcare resource utilization plays a key role in determining TAVR’s financial impact. Studies indicate that TAVR reduces overall hospital readmissions compared to SAVR, particularly by lowering rates of heart failure rehospitalization and reintervention [16]. A nationwide study in Korea, analyzing 2175 TAVR patients, found that post‐TAVR medical costs decreased significantly in both intermediate‐frailty and high‐frailty patients [17]. Moreover, all frailty groups had significantly shorter hospital stays post‐TAVR, reinforcing the notion that TAVR leads to cost savings by reducing the overall burden on healthcare systems. However, concerns persist regarding post‐TAVR conduction disturbances, which often necessitate PPI in up to 15% of cases [14]. While PPI is considered a minor complication in the short term, it introduces long‐term costs related to device follow‐ups and replacements, which should be factored into economic assessments. Health systems with universal healthcare models are incorporating TAVR into national guidelines for low‐ and intermediate‐risk patients [15]. However, disparities in TAVR access across healthcare settings remain a concern, as evidenced by readmission patterns largely driven by geographic barriers [18].

### 4.2. Strengths

The present study has several notable strengths. First, it adheres strictly to the VARC‐3 criteria for defining clinical outcomes, ensuring that the reported results are standardized and comparable to international registries. Second, beyond standard clinical outcomes, this study provides a unique comparison of nursing and care management strategies (Table 6) between TAVR and SAVR for pure AR. This practical perspective offers actionable insights for heart teams optimizing perioperative protocols for distinct patient groups.

### 4.3. Future Implications

As TAVR technology matures, its indication is likely to expand further into the low‐risk, younger AR population. The findings suggest that while TAVR is safe, future device iterations must focus on reducing conduction disturbances and paravalvular leakage to compete with the long‐term durability of surgical valves. From a healthcare policy perspective, reimbursement models should be adjusted to account not just for the procedural cost, but for the savings associated with reduced mechanical ventilation duration and faster physical recovery. Large‐scale, randomized controlled trials are warranted to establish long‐term guidelines for this specific demographic.

### 4.4. Limitations

This study has several limitations. First, as a retrospective analysis, it relies on preexisting data, which may introduce information bias due to inconsistent variable measurement. Additionally, unrecorded confounders could limit the accuracy of adjustments. Second, the small sample size reduces statistical power, restricting the ability to detect clinically significant differences and limiting the generalizability of findings. Lastly, the absence of long‐term follow‐up data prevents a comprehensive assessment of disease progression and long‐term outcomes. Future research should focus on larger sample sizes and prospective randomized trials to enhance reliability and applicability.

## 5. Conclusion

In conclusion, TAVR serves as a safe and effective alternative to SAVR in selected low‐ to intermediate‐risk patients with pure AR. While TAVR offers significant advantages in terms of operative efficiency and reduced mechanical ventilation, it presents a unique profile of complications, particularly conduction abnormalities, which differ from the heart failure and delirium risks associated with SAVR. These findings underscore the necessity for procedure‐specific perioperative management strategies. As the indications for TAVR expand to low‐risk populations, a multidisciplinary heart team approach remains essential to optimize patient selection and long‐term outcomes.

## Funding

This study was funded by a research grant of the National Key Research and Development Program (2023YFC2412400), Ministry of Science and Technology of the People’s Republic of China.

## Conflicts of Interest

The authors declare no conflicts of interest.

## Data Availability

The data that support the findings of this study are available from the corresponding author upon reasonable request.
